# A True Bilateral Superficial Femoral Artery Aneurysm: A Case Report

**DOI:** 10.7759/cureus.49599

**Published:** 2023-11-28

**Authors:** Shahad Abdulkhaleq Mamalchi, Dhafer M Kamal

**Affiliations:** 1 College of Medicine, Royal College of Surgeons in Ireland - Medical University of Bahrain, Manama, BHR; 2 Department of Surgery, Bahrain Defence Force Royal Medical Services, Military Hospital, Riffa, BHR

**Keywords:** bilateral aneurysms, femoral artery aneurysm, bilateral sfa aneurysms, rare aneurysms, superficial femoral artery, superficial femoral artery aneurysm

## Abstract

A 67-year-old male of Egyptian descent presented to the vascular outpatient clinic with a left lower limb pulsating mass in the mid-inner thigh region. A computed tomography angiography (CTA) revealed a 3x4cm left mid-superficial femoral artery (SFA) aneurysm and a 2x3cm aneurysm in the right mid-SFA. An open repair of the left, followed by right SFA aneurysms, was performed in a sequential matter, six weeks apart. The patient’s recovery and follow-up were uneventful. Pathology revealed both specimens to be true atherosclerotic aneurysms. This is a case report of a true bilateral SFA aneurysm.

## Introduction

True aneurysms of the superficial femoral artery (SFA) are rare and often diagnosed late due to their anatomical location within the thigh [1]. Patients, being mostly of the elderly male population, may present with a pulsating mass in the thigh, pain, venous compression, or be asymptomatic [2]. Twenty-six percent of true SFA aneurysms are bilateral and are commonly associated with synchronous peripheral artery aneurysms [2]. Popliteal or common femoral artery aneurysms are usually preceded by embolization or thrombosis, whereas the most common complication of SFA aneurysms is rupture [3]. In this case report, we present a true bilateral SFA aneurysm, which was initially presented on the left side, in an elderly male of Egyptian descent.

## Case presentation

A 67-year-old male, with a known case of coronary artery disease, dyslipidemia, and a former 40-pack-year cigarette smoker, presented with a pulsating mass on the inner aspect of the left thigh. This was noticed by the patient three weeks prior to presentation. The mass was otherwise asymptomatic. On examination, a non-tender mass, measuring around 4x4 cm in size, with an expansile pulse, was found in the middle, inner region of the left thigh. All peripheral pulses were palpable. There is no history of alcohol or recreational drug usage, pyrexia, oral or genital ulcers, and no needle prick sites were found.

Our patient has no history of trauma or inflammatory symptoms or signs. Moreover, there is no history of connective tissue diseases, such as Ehlers-Danlos syndrome, Marfan’s syndrome, or Behçet's disease. The patient's family history revealed that his father had suffered from a ruptured popliteal artery aneurysm, which was surgically repaired.

**Figure 1:**
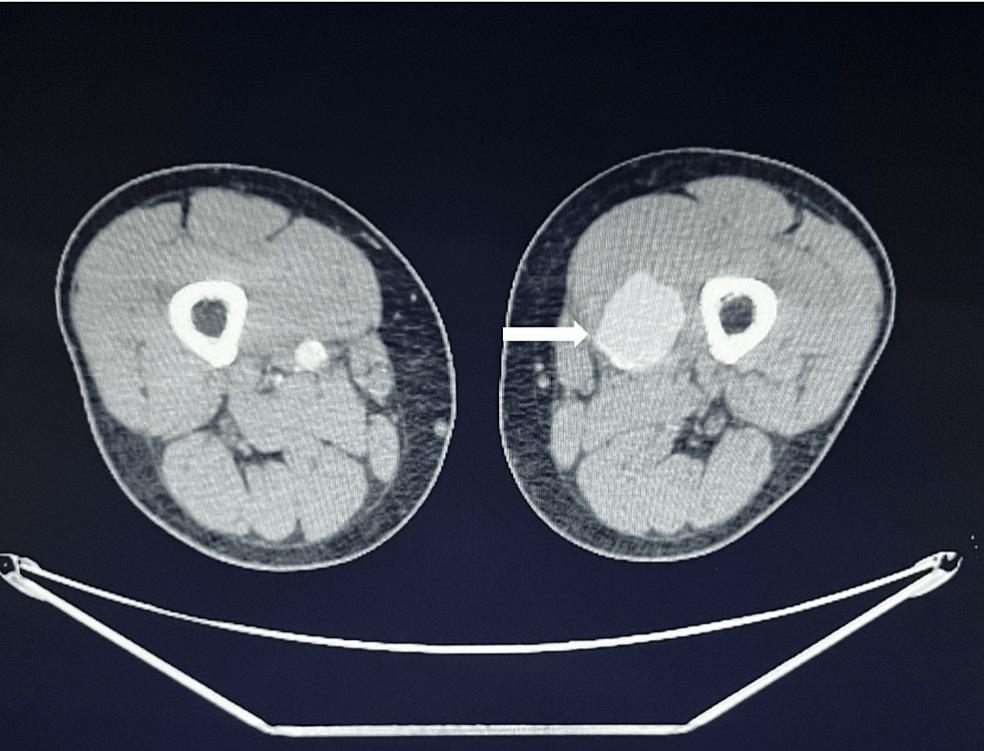
CTA revealing the left-sided superficial femoral artery aneurysm with dimensions of 4x4cm. CTA: Computed tomography angiography

**Figure 2:**
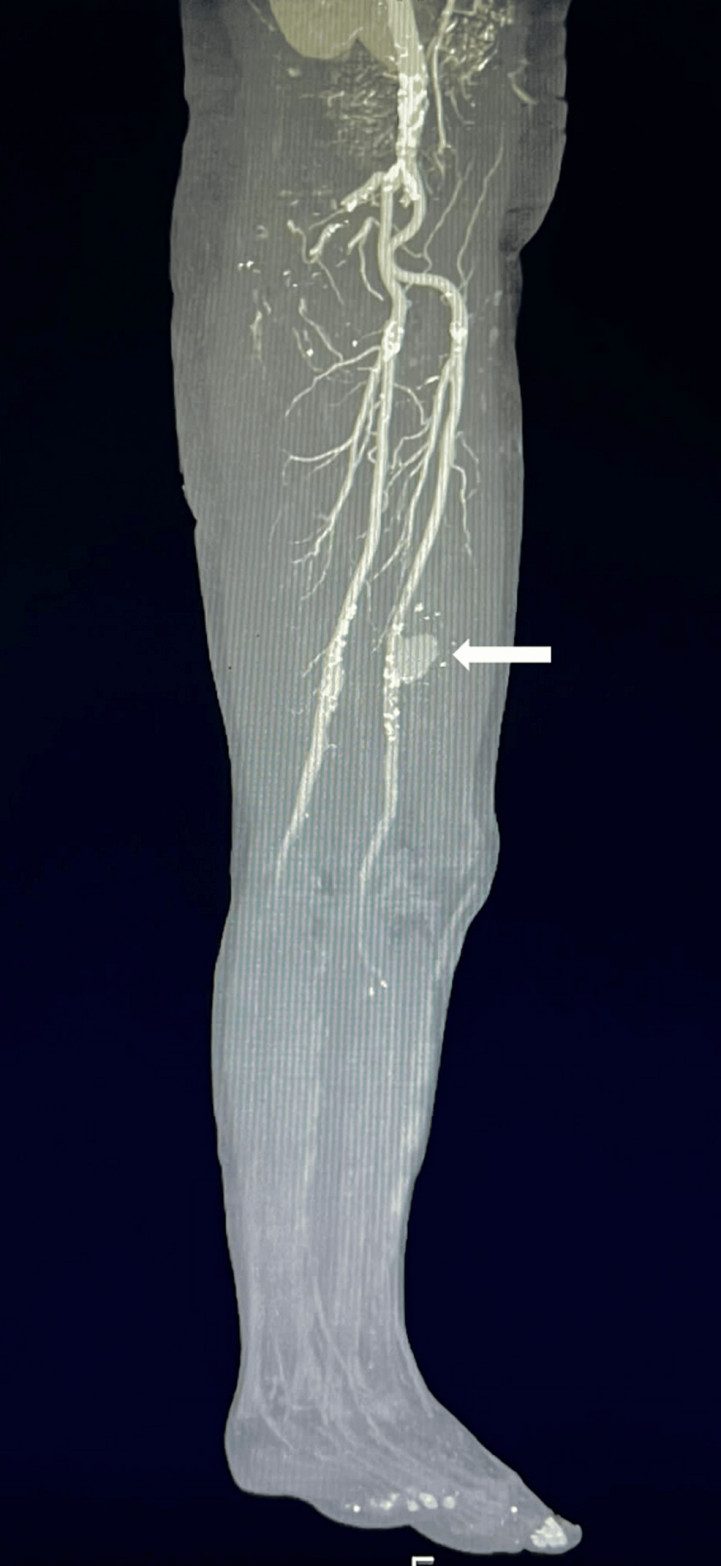
CTA revealing the left-sided saccular superficial femoral artery aneurysm protruding anteriorly. CTA: Computed tomography angiography

**Figure 3:**
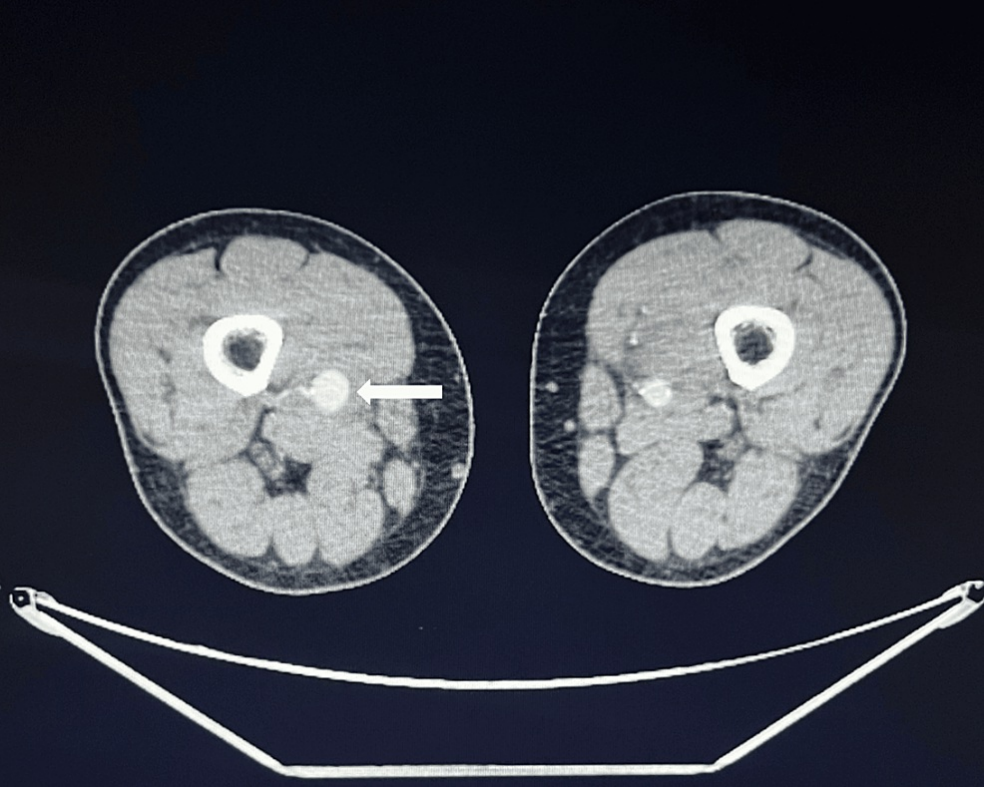
CTA revealing the right-sided superficial artery aneurysm with dimensions of 2.5x3.5cm. CTA: Computed tomography angiography

Investigations involved laboratory work, serology, and radiology. Some etiologies of SFA aneurysms are Behçet’s disease, syphilis, and Wegener’s granulomatosis [1]. Rheumatological serology was negative, and the patient had no history of painful mouth ulcers, genital ulcers, or eye inflammation; therefore, Behçet’s disease was unlikely. Treponemal and non-treponemal serology tests were negative, ruling out syphilis. Further blood work results included normal red blood cell (RBC) count, white blood cell (WBC) count, hemoglobin (Hb), and mean corpuscular volume (MCV). Relevant antibodies, such as antineutrophil cytoplasmic antibodies (ANCA), were negative; therefore, Wegener's granulomatosis is also unlikely. Arterial duplex and CT/angio confirmed the presence of a 3x4 cm mid-left SFA aneurysm as seen in Figure 1, mostly saccular in shape and protruding anteriorly, as seen in Figure 2. It also depicted a right SFA fusiform aneurysm 2x3 cm in diameter, as seen in Figure 3. The wall of the aneurysm is clearly visible on the CTA. The entire aorta and visceral arteries were free from aneurysms.

Both femoral artery aneurysms warrant repair due to their large size and the risk of rupture or distal embolization. Open surgical and endovascular repairs are both available options for treatment, with the former more suitable for lower-risk patients and the latter more suitable for higher-risk patients. We feel the open repair, although has a higher risk of peri-operative morbidity, is a more durable long-term solution for this problem and was suitable for our patient. The left SFA aneurysm was treated first due to its large diameter and higher risk for rupture. The procedure was carried out under general anesthesia via a direct approach over the aneurysm, which was resected and replaced with an interposition graft of the ipsilateral reversed great saphenous vein. The postoperative recovery was uneventful. Pathology confirmed a 3x4cm aneurysm consistent with the atherosclerotic type.

The right SFA aneurysm was successfully repaired in the same manner six weeks later.

The patient was followed up regularly at the vascular clinic for 30 months. The findings of acute brachial index (ABI), toe brachial index (TBI), and transcutaneous oxygen pressure (TcPO2) tests remain normal. His lower limbs remain well-perfused as confirmed by the presence of distal pulses and patent bypass grafts on arterial duplex with normal flow velocities and no evidence of anastomotic stenosis nor aneurysm recurrence.

## Discussion

SFA aneurysms are uncommon, and the occurrence of bilateral SFA aneurysms is quite rare. They continue to be infrequent in comparison to aneurysms found in adjacent arteries like the popliteal and common femoral arteries [1]. This infrequency is thought to be attributed to the specific anatomical positioning of the SFA within Hunter's canal, where the artery is shielded by the surrounding musculature and minimal bending stress [1]. In American men, the reported incidence of femoral artery aneurysms is 7.39 per 100,000 individuals [4]. When considered in isolation, SFA aneurysms constitute only 0.5% of all peripheral aneurysms and 1% of all femoral artery aneurysms [5,6]. It is worth noting that these studies may have limitations due to variations in patient demographics and the imaging techniques utilized.

Early diagnosis of SFA aneurysms is important to minimize adverse outcomes [1]. Among the potential complications are rupture, infection, and limb ischemia due to distal embolization or thrombosis [1]. SFA aneurysms are symptomatic on presentation in 63% of cases [5]. Elderly patients, with 87% being male, usually present with pain and a pulsatile mass in the thigh, indicating a symptomatic aneurysm, with or without rupture [7]. Typically, aneurysms occur in the middle to lower third of the artery [7]. The average diameter at diagnosis is 8.4cm, and if larger than 2.5cm in size, repair must be done to avoid complications, poor outcomes, and limb loss [7]. However, as a retrospective study by Lawrence et al. argues, the “size” criterion is outdated, and thanks to advanced imaging modalities, other factors may influence the decision to intervene, such as etiology, character of intraluminal thrombus, and less invasive repair methods [7,8].

Our patient presented clinically with a left SFA aneurysm; however, a synchronous aneurysm was found in the right SFA on imaging. For this reason, the surgeon should look for other synchronous aneurysms. These investigations must be repeated at short- and long-term follow-up. Particularly, CTA should be the first-line investigation and diagnostic tool as it reveals key components for diagnosis, such as anatomic location (shape, length, and diameter) and pre-operative planning [9]. Determination of etiology is important for definitive management. The following causes of SFA aneurysms must be ruled out: syphilis, Wegener’s granulomatosis, Behçet's disease, inflammatory conditions, and connective tissue diseases such as Ehlers-Danlos syndrome or Marfan’s syndrome [1].

The two options for surgical treatment of SFA aneurysms are open and endovascular repair. Open repair has favorable short-term and long-term outcomes with superior limb salvage [10]. Secondary interventions for limb salvage are more frequent when endovascular repair is performed for these aneurysms [10]. Choosing between open and endovascular repair also depends on the individual patient’s comorbidities, physical mobility, social support, and independence [10]. If arteriovenous fistula, infection, or ischemic changes are present, open repair becomes the preferred first-line treatment [10]. Most cases using endovascular repair reported in the literature do not provide data on long-term patency and follow-up.

## Conclusions

Bilateral SFA aneurysms are rare. They are commonly found in the middle and distal segments of the artery. Duplex ultrasonography (DUS) and CTA are useful imaging tools to confirm diagnosis and plan management. Early and prompt diagnosis and repair are limb-saving and potentially life-saving. Both open and endovascular repair are available options for the definitive management of these aneurysms. Our patient was diagnosed and treated in a timely fashion. An open repair of both SFA aneurysms was successfully undertaken in a sequential manner with excellent results confirmed initially and up to 30-month follow-up.
